# Colostrum management practices that improve the transfer of passive immunity in neonatal dairy calves: A scoping review

**DOI:** 10.1371/journal.pone.0269824

**Published:** 2022-06-29

**Authors:** T. Uyama, D. F. Kelton, C. B. Winder, J. Dunn, H. M. Goetz, S. J. LeBlanc, J. T. McClure, D. L. Renaud

**Affiliations:** 1 Department of Population Medicine, Ontario Veterinary College, University of Guelph, Guelph, ON, Canada; 2 Department of Health Management, Atlantic Veterinary College, University of Prince Edward Island, Charlottetown, PE, Canada; Tokat Gaziosmanpasa Universitesi, TURKEY

## Abstract

The objective of this scoping review was to describe the literature on the characteristics and management practices of colostrum feeding and their associations with the level of transfer of passive immunity (TPI) in dairy calves. Observational and experimental studies were searched in 5 electronic databases and 3 conference proceedings. Two reviewers independently screened primary studies, either analytic observational or experimental studies written in English. Studies on dairy or dual-purpose calves with passive immunity analyzed by blood sampling between 1 to 9 days of age were included. All studies had to compare at least one colostrum intervention or risk factor and their association with passive immunity. Of the 3,675 initially identified studies, 256 were included in this synthesis. One hundred and ninety-five were controlled trials, 57 were cohort studies, and 4 were cross-sectional studies. The effect of colostral quantity at first feeding was investigated in 30 controlled studies including studies that were comparable to each other. The effect of colostral quality was explored in 24 controlled studies with inconsistent criteria used to define the quality. The effect of the timing of first feeding of colostrum was investigated in 21 controlled studies, where the timing of feeding ranged widely from immediately after birth to 60 h of age. Only 4 controlled studies evaluated the relationship between bacterial load in the colostrum and TPI in dairy calves. Of the 256 total studies, 222 assessed blood IgG concentration while 107 measured blood total protein concentration. We identified a gap in knowledge on the association between passive immunity in dairy calves and the bacterial load in colostrum, or the timing of harvesting colostrum from the dam. A possible quantitative synthesis could be conducted among the studies that evaluated colostral quantity at the first feeding in relation to TPI in dairy calves.

## Introduction

Colostrum is the first milk harvested following calving [1] and is an important source of immunoglobulins (Ig) which enhance the immune system of calves. In addition, colostrum contains greater levels of protein, fat, hormones, minerals, and vitamins compared to whole milk. Poor quality or management of colostrum feeding can result in failed transfer of passive immunity (FTPI), most commonly defined as serum IgG concentration < 10 g/L in dairy calves at 24 to 48 h after birth [1]. In a meta-analysis study, calves with FTPI have twice the risk of dying, 1.8 times greater risk of developing respiratory disease, and 1.5 times greater risk of having diarrhea compared to those without FTPI [2]. Therefore, excellent colostrum management is vital to calf health and welfare.

The main components of successful transfer of passive immunity are to provide an adequate amount of high-quality colostrum to dairy calves as soon as possible after birth. Researchers recommend focusing on quantity, the interval from calving to first feeding of colostrum, colostral quality (high quality: IgG > 50 g/L), and cleanliness of colostrum (i.e., low bacterial load in the colostrum) [1]. Studies have found inconsistent results in relation to the quantity, the timing of feeding colostrum, or dam parity in associations with the level of transfer of passive immunity (TPI) in calves [3–5], which could be explained by the differences in study designs, populations, definitions on the risk factors, predictors and covariates included in the model, or methods of measuring the level of TPI. In addition, other factors may be important for successful transfer of passive immunity, including calving season, pre-parturient diet in dry cows, and dam breed that may associate with colostral quality [1]. Hence, it is important to frame the quantity and characteristics of the published literature on these variables.

A scoping review summarizes the extent and characteristics of the literature on a research question to answer a broader topic than the one developed for a systematic review [6]. A scoping review can be a precursor to a systematic review and may reveal knowledge gaps [6]. The objective of this scoping review was to describe and characterize the literature investigating the characteristics of colostrum and its management related to the level of TPI in dairy calves. The number of explanatory variables of interest, detailed below, underscores the need for a synthesis of the information available.

## Materials and methods

Arksey and O’Malley [6] and the Preferred Reporting Items for Systematic Reviews and Meta-Analysis extension for Scoping Reviews (PRISMA-ScR) [7] served as reporting guidelines for this review. Prior to conducting the search, a protocol was developed and archived in the institutional repository of the University of Guelph (http://hdl.handle.net/10214/17970). Any deviations to the protocol are described in the manuscript.

### Eligibility criteria

Eligible studies were those with full texts available in English. Case reports, case series, and non-primary studies (i.e., review papers) were excluded. Analytic observational studies and experimental studies were eligible, including controlled trials, cohort studies, case-control studies, and cross-sectional studies. Studies on dairy or dual-purpose calves, either male or female, were included; those on beef calves were excluded. There were no restrictions on management type, for example, studies on organic farms and farms with seasonal calving were included. Conference proceedings were included if the abstract had at least 500 words.

Eligible interventions or risk factors related to colostrum practices were categorized based on Godden et al. [1]. These factors were: 1) dam age or parity; 2) dam breed; 3) pre-parturient nutrition; 4) calving season; 5) pre-parturient vaccination; 6) dry period length; 7) volume of colostrum produced at the first milking following calving; 8) timing of colostrum harvest; 9) calving difficulty; 10) hypoxia or acidosis in calves; 11) non-separation between dam and calves; 12) timing of first colostrum feeding; 13) colostrum quality as measured in relation to IgG concentration; 14) quantity of colostrum at first feeding; 15) total quantity of colostrum fed in 24 h; 16) extended feeding of colostrum or transition milk (i.e., second to 6^th^ milkings postpartum) after 24 h of age; 17) source of colostrum (e.g., colostrum replacer (CR), colostrum supplement (CS), or pooled colostrum); 18) heat treatment of colostrum; 19) bacterial load in colostrum; 20) storage method for colostrum; and 21) route of colostrum feeding. Studies were included when populations were compared at different levels of at least one of the aforementioned factors. Studies that measured the risk factors at the herd level were excluded to avoid ecological fallacy.

Eligible outcomes included any one of the following methods of assessing calf serum or blood IgG concentration, total protein (TP) concentration, Brix percentage, or gamma-glutamyl transferase (GGT) concentration measured in calves 1 to 9 d of age after exposure to a risk factor or intervention [8].

### Information sources and search strategy

Literature was searched through Medline (via Ovid), CAB Direct (via CABI), Scopus, Agricola (via ProQuest), ProQuest dissertation and theses, Science Citation Index Expanded (SCI-EXPANDED), Conference Proceedings Citation Index—Science (CPCI-S), and Emerging Sources Citation Index (ESCI) (via Web of Science). No limitation on year of publication was set before the search unless the database had its own limitation. Grey literature was searched manually through the conference proceedings between 1997 and 2020 of the American Dairy Science Association, World Buiatrics Congress, and the American Association of Bovine Practitioners as they were the major international conferences conducted in English. Electronic databases were searched on June 21 and 22, 2020, and the grey literature between Oct 29 and Nov 13, 2020.

Search terms were developed with a combination of terms related to the study population, colostrum, and the outcomes (Table 1). These search terms were validated by identifying 25 pre-selected studies. The search string was adjusted for each database.

**Table 1:** Search strings used to collect studies related to colostrum characteristics and management and their relation to transfer of passive immunity in dairy calves.

	Search terms
1	calf OR calves OR heifer OR bull OR female OR male OR veal
2	colostr* OR “first milk”
3	1 AND 2
4	Immunoglobulin OR IgG* OR “passive transfer” OR FPT OR TP OR “total protein” OR “antibody deficiency” OR “passive immune” OR “passive immunity” OR Brix
5	3 AND 4

### Study selection

All studies were imported and de-duplicated in EndNote X7 (Clarivate Analytics, Philadelphia). The studies were exported, stored, and further de-duplicated on Distiller Systematic Review (DSR) Software (Evidence Partners Inc., Ottawa). All screening processes and data extraction were performed on DSR.

### Screening processes

Screening questions were developed to capture all relevant studies that met the inclusion criteria. Title and abstract screening were performed with the following questions:

1. Is the title or abstract written in English?
2. Does the title or abstract describe a primary research study?
3. Does the title or abstract or both include results of serum or blood IgG (total IgG or IgG_1_), TP, Brix %, or GGT in calves?

The title and abstract screening questions were pre-tested for 100 studies among 5 reviewers and any adjustments to the screening questions were made for clarity. All questions were answered as “yes”, “no”, or “unclear”, and the studies with “yes” or “unclear” were retrieved and included in the full-text screening.

Full-text screening was performed with the following questions:

1. Is the full article written in English?
2. Is the full article available?
3. Does the full article describe a primary research study?
4. Does the full article include results of serum or blood IgG (total IgG or IgG_1_), TP, Brix %, or GGT in either dairy, dual-purpose, or veal calves?
5. Was the study either a randomized controlled trial, non-randomized experimental study, cohort study, case-control study, or a cross-sectional study with a risk factor at calf level?
6. Does the study compare at least one of the characteristics below? (age or parity of the dam, breed of the dam, nutrition during the pre-parturient period, season of calving (include heat or cold stress), vaccination during the pre-parturient period, dry period length, volume of colostrum produced in the first milking after calving, timing of collecting colostrum after calving, delivery difficulties, hypoxia or acidosis in calves after birth, housing the calf together with its dam, timing of the first feeding of colostrum after birth, quality of colostrum, quantity of colostrum at first feeding, total quantity of colostrum fed within 24 h after birth, extended feeding of colostrum or transition milk after 24 h of birth, source of colostrum, heat treatment of colostrum, bacterial load in colostrum, storage of colostrum, or route of feeding colostrum)
7. Was the outcome measured after feeding the colostrum?
8. Was the outcome measured in calves 1 to 9 d of age?

The full-text screening questions were pre-tested for 25 studies among 5 reviewers and any amendments on the screening questions were made. All questions were answered as “yes” or “no.” Studies with “no” for any question were removed at that level with the reason of exclusion at full text noted. Those that received a “yes” for all questions were eligible for data extraction and synthesis.

At both title or abstract screening and full-text screening, 2 reviewers independently answered the screening questions. When the reviewers had conflicting answers, they resolved the conflict through discussion, or a third reviewer was resolved the conflict with a deciding vote.

### Data extraction

Information on the characteristics of each study was extracted independently by 2 reviewers from all the studies that passed through the screening questions and met the inclusion criteria. To extract information from each study, a data extraction form was developed and pretested in 10 studies by 5 reviewers. Extracted data were validated between 2 reviewers and a third reviewer served as mediator when a conflict remained. Data extraction was performed to collect the following information for each study:

1. General information on the study (publication year, country where the study was conducted, study period, objective, hypothesis)
2. Study population (calf breed, sample size, calf sex, calf production type)
3. Study design (controlled trial, cohort, case-control, cross-sectional study)
4. Risk factor or intervention that was assessed and the covariates
5. Outcome (IgG, TP, Brix %, GGT), method of outcome measurement, timing of blood sampling, the cut-off used to define FTPI and whether the proportion of calves with FTPI was measured

### Data synthesis and charting process

All the above information that was extracted from the eligible studies were exported from DSR to a Microsoft Excel spreadsheet (Microsoft Corp. Redmond, WA, USA). Descriptive statistics were generated in Microsoft Excel and the proportions, the numbers of studies, and their characteristics were summarized in tables and figures.

## Results

**Fig 1:**
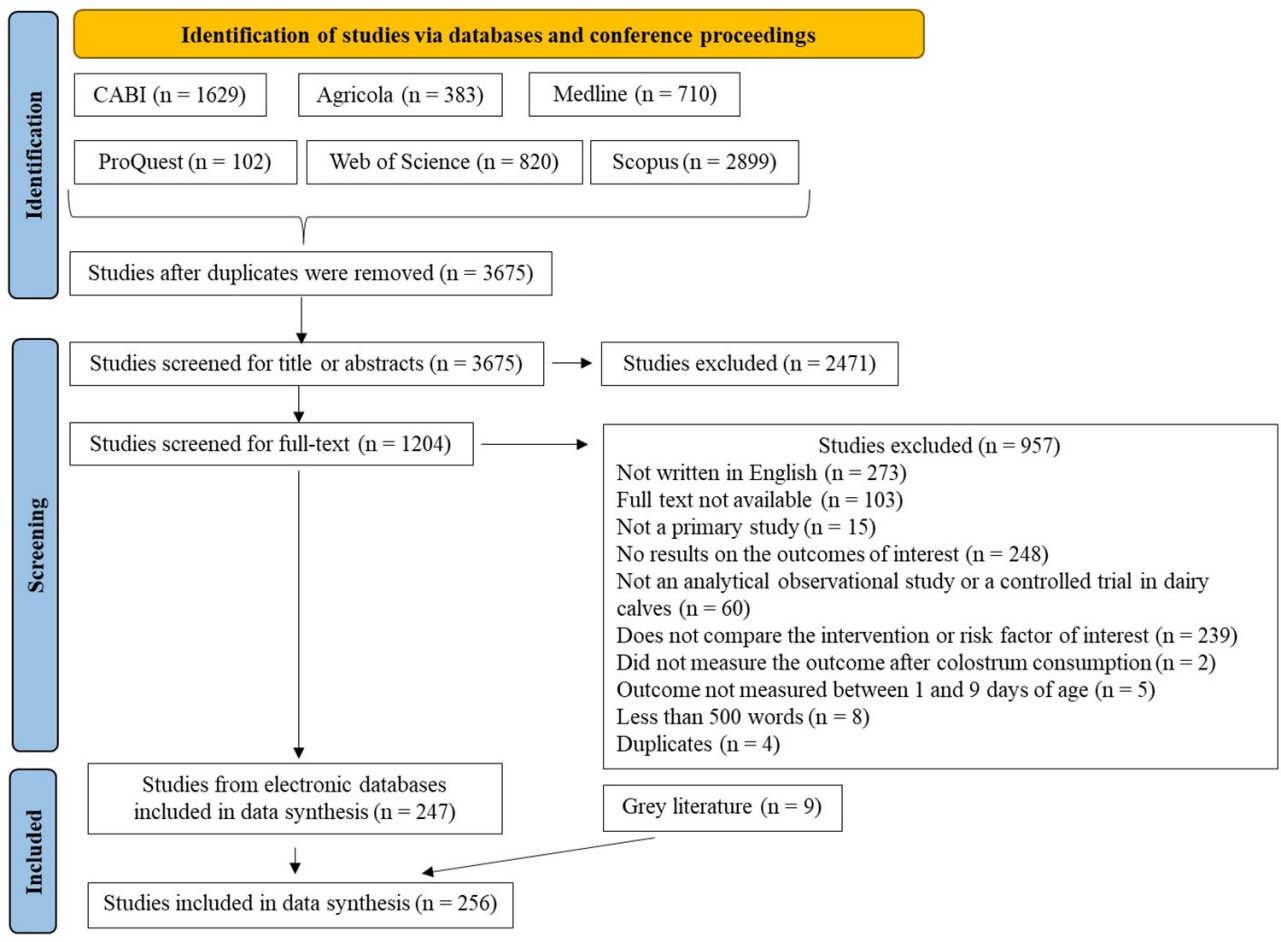
Flow chart of studies identified, screened, and included in the data analysis [30].

A total of 3,675 studies were identified through electronic databases after de-duplication and their titles and abstracts were screened, leading to the exclusion of 2,471 studies. The remaining 1,204 studies were screened at the full-text level and 957 were excluded for the reasons shown in Fig 1. Nine studies were identified in the grey literature. In total, 256 studies were included in data extraction and synthesis.

### Study demographics

**Fig 2:**
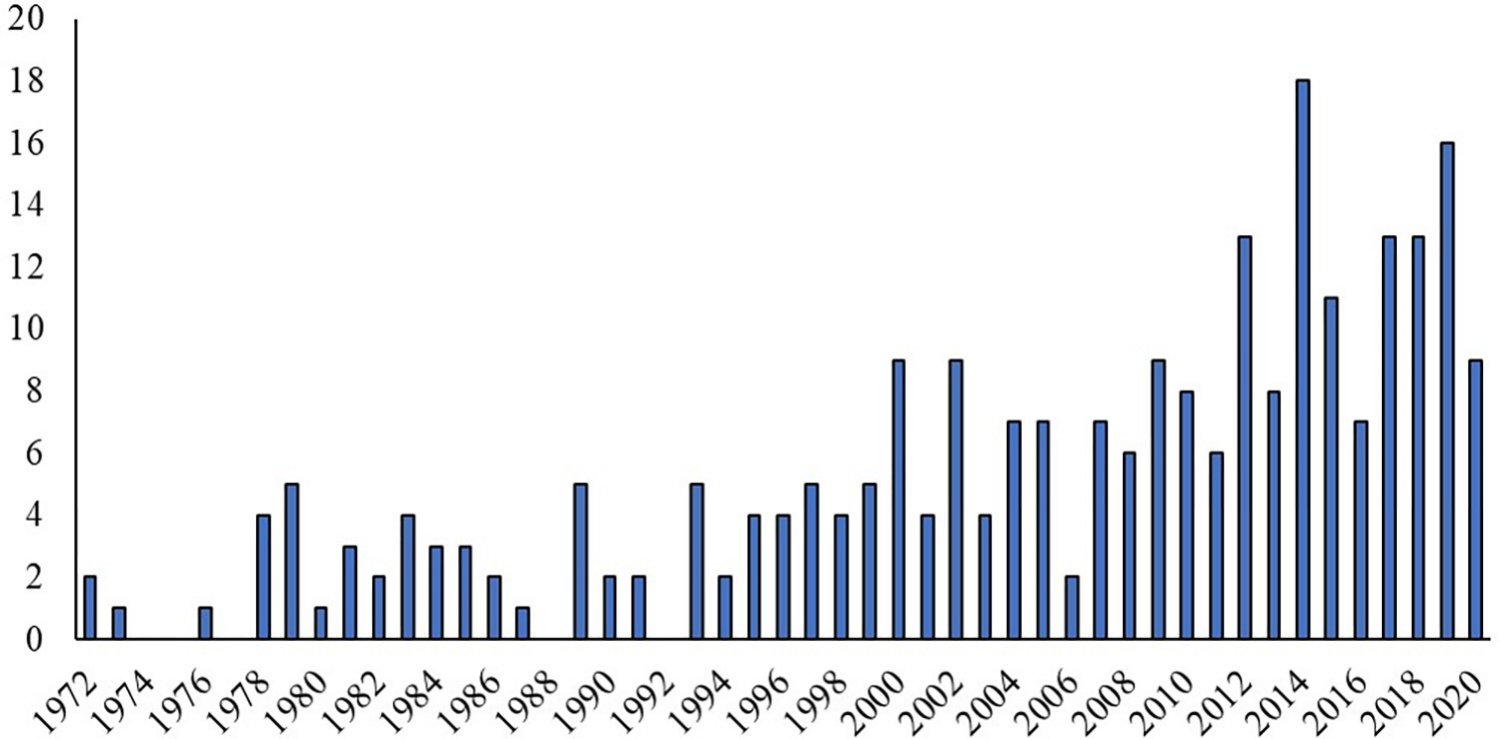
The number of studies on colostrum characteristics and management practices on transfer of passive immunity in dairy calves published each year between 1972 and 2020 (n = 256).

The studies included in data synthesis were published between 1972 and 2020 with nearly half (48%; n = 122) being published from 2010 onwards (Fig 2). Most studies (59%; n = 151) did not report the year the study was conducted, 10% (n = 25) were done before 2000, 13% (n = 33) in 2000 to 2010, and 18% (n = 47) in 2011 to 2020. Most of the studies were performed in North America (39%; n = 101) and Europe (14%; n = 37); however, the country where the study was conducted was not reported in 35% of studies (n = 90). Most (78%; n = 199) of the studies did not state a hypothesis and 2% (n = 4) did not state an objective.

Three-quarters of studies (76%; n = 195) were controlled trials, followed by cohort studies (22%; n = 57) and cross-sectional studies (2%; n = 4). Most (59%; n = 152) studies used Holstein calves, followed by 9% (n = 23) using cross-bred calves, 7% (n = 17) using Jersey calves, and 4% (n = 11) using other dairy breeds. Thirty-six percent (n = 91) of included studies did not report the breed of the calves. Thirty percent (n = 76) used female and male calves, 18% (n = 46) used male calves, 15% (n = 38) used female calves, and 38% (n = 96) did not report the sex used. Most studies (75%; n = 192) used dairy calves; however, 25% (n = 64) did not report the production type of calves. Many studies that did not report the calf production type were eligible for this review as 44 studies used calves of dairy breeds or crossbreds of dairy breeds or dual-purpose breeds, 11 studies used calves born from dairy breeds, 8 studies used calves fed colostrum by caretakers, and 1 study used calves of a dual-purpose breed.

**Fig 3:**
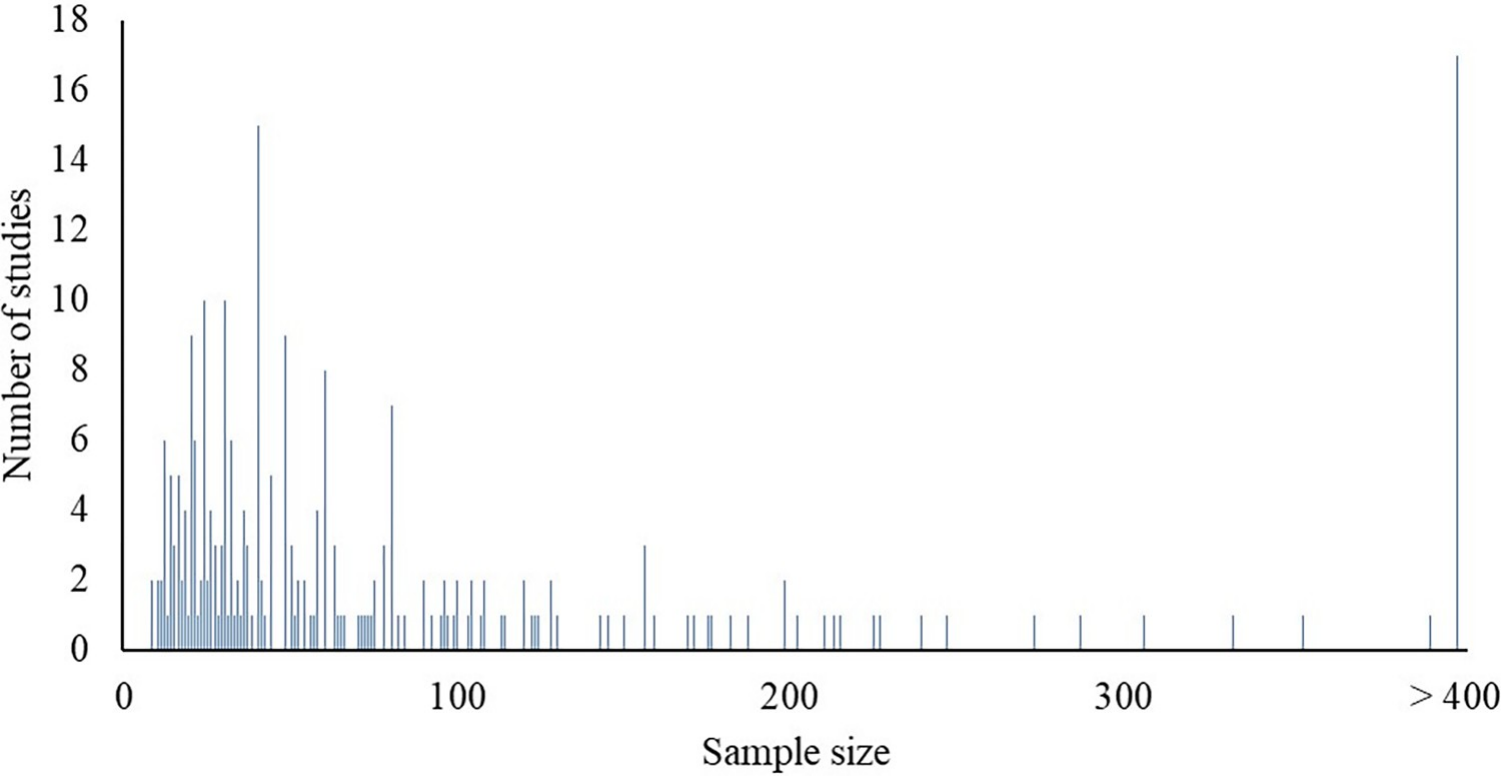
Distribution of the sample size used in studies (n = 254). Two studies had unclear or did not report the sample size.

The sample size was extracted as the number of calves initially enrolled in the study. When the number of calves was not reported (n = 14), the number of dams was applied as a substitute. Sample size ranged from 8 to 3,819 calves, with 75% (n = 192) using 8 to 100 calves, 12% (n = 31) using 101 to 200 calves, 7% (n = 18) using 201 to 500 calves, 2% (n = 4) using 501 to 1000 calves, and 4% (n = 9) using 1001 to 3819 calves, as shown in Fig 3. One study did not report the sample size, and 1 study had an unclear sample size.

Among the 195 controlled trials, 83% (n = 161) did not adjust for a covariate in model building. The common variables adjusted as a covariate was the age or parity of the dam (n = 18), the timing of first feeding of colostrum (n = 13), calving difficulty (n = 11), colostral quality (n = 11), the quantity of colostrum fed at first feeding (n = 7), dam breed (n = 3), the amount of colostrum produced by the dam (n = 3), and the total quantity of colostrum fed within 24 h of birth (n = 3).

### Interventions and risk factors evaluated

Total of 256 studies included were categorized into each risk factor or intervention investigated as shown in S1 Table (https://doi.org/10.5683/SP3/NUOT4P)). Among the 195 controlled trials, 146 included one intervention and 49 included multiple interventions. Among the 57 cohort studies, 25 studies included one risk factor and 32 included multiple risk factors. All 4 cross-sectional studies included multiple risk factors.

#### Quantity of colostrums

**Fig 4:**
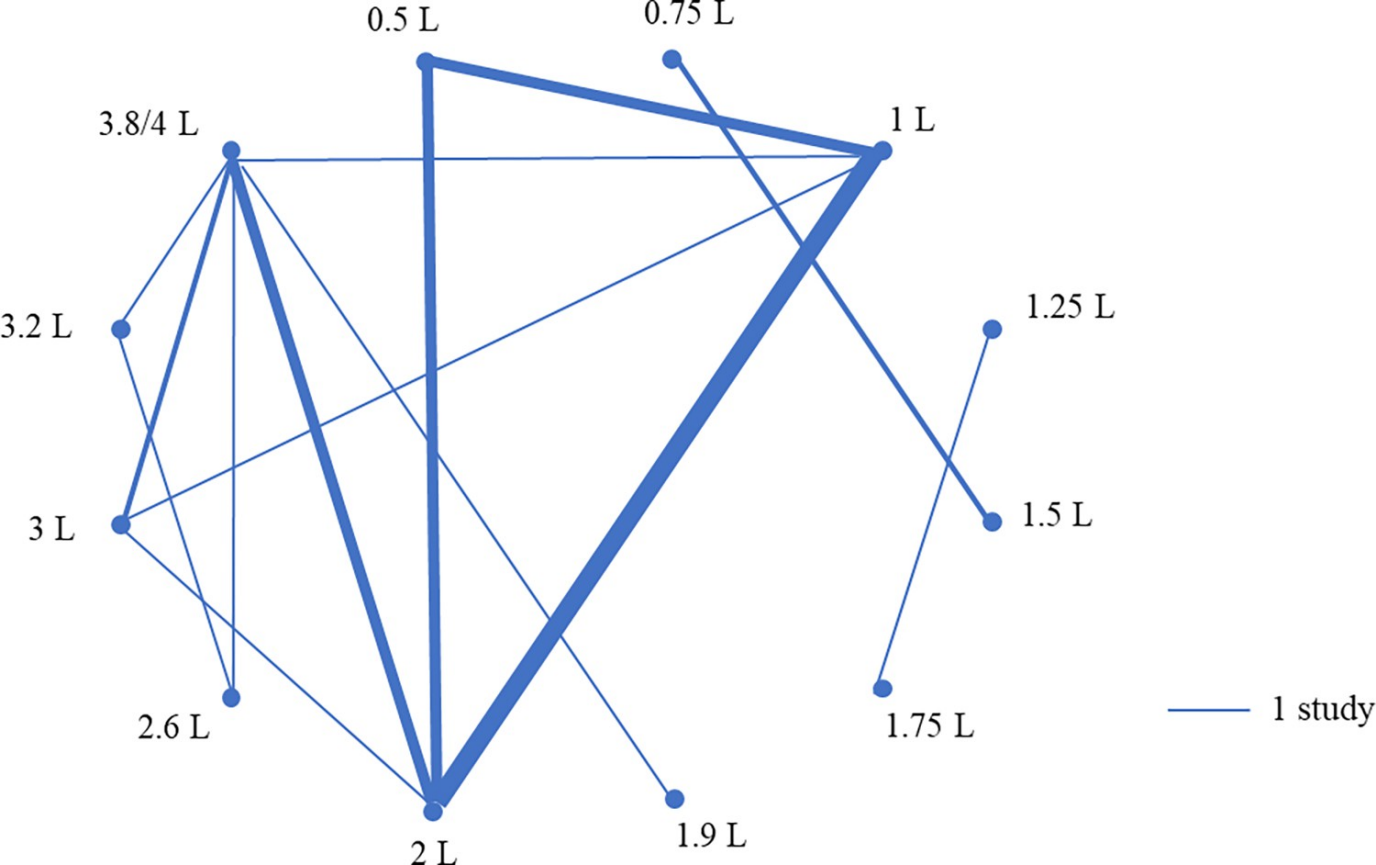
Schematic summary of the volumes of colostrum compared in 17 controlled studies that compared different quantities of maternal colostrum fed at first feeding. Quantity fed at a proportion of body weight was converted to liters based on the average body weight reported in 2 studies. The thickness of lines represents the number of studies conducted between the combinations of treatment groups.

Thirty controlled trials assessed the quantity of colostrum fed at first feeding (S2 Table: https://doi.org/10.5683/SP3/NUOT4P). Thirteen studies evaluated the effect of colostral quantity by adjusting the number of packages or the amount of colostrum product fed to calves and the other 17 studies controlled the allocation of different volumes of maternal colostrum fed to calves. Among these latter studies, the number of studies and treatment groups are shown in Fig 4. Nine cohort studies assessed the quantity of colostrum at first feeding, where 5 assessed the quantity as a continuous variable, and other studies categorized the quantity of first feeding in 0.5 or 1 L increments. One cross-sectional study investigated the quantity of the first feeding either below or above 3.78 L.

### Total quantity of colostrum fed within 24 h of birth

Eight controlled trials assessed the total quantity of colostrum fed within 24 h of birth, including 2 which compared 1.5 L to 3 L, 1 compared 1.5 L, 3 L, or 4.5 L, 1 compared 3.2 L to 5.2 L, 1 compared 4 L to 6 L, 1 compared 4 L to ad libitum, 1 compared 6 L to 8 L, and 1 compared 1 to 2 meals of CR (S3 Table: https://doi.org/10.5683/SP3/NUOT4P). Two cohort studies assessed the total quantity of colostrum fed in 24 h as a continuous variable. Two cross-sectional studies assessed the total quantity of colostrum fed in 24 h including 1 describing it as the number of colostrum feedings and 1 categorizing it as < 3.9 L, 3.9 to 5.0 L, 5.1 to 5.9 L, and > 6 L.

### Colostrum quality

**Fig 5:**
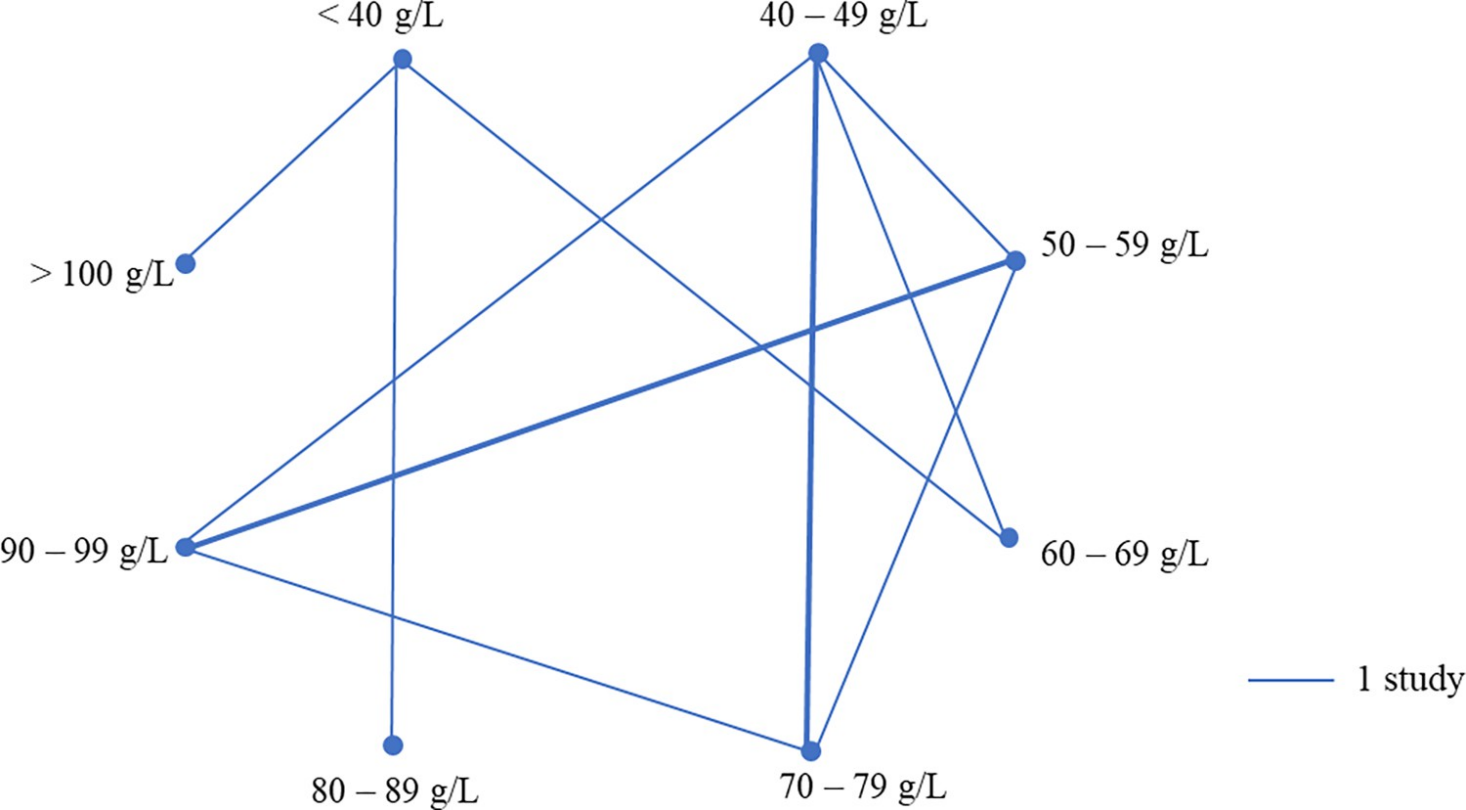
Schematic summary of the concentrations of IgG in colostrum in 24 controlled studies that compared colostral quality. Studies that targeted other risk factors as the main intervention (n = 10), wider range of colostral Ig concentration (n = 4), no information on colostral Ig concentration (n = 2) and fermented compared with unfermented colostrum (n = 1) were excluded. The thickness of lines represents the number of studies conducted between the combinations of treatment groups.

Twenty-four controlled trials assessed the quality of colostrum based on its Ig concentration (S4 Table: https://doi.org/10.5683/SP3/NUOT4P), where 14 controlled the allocation of different quality of colostrum fed to calves, and the other 10 studies investigated colostrum quality as a secondary outcome and assessed its relationship with the outcome. Among the 14 studies that allocated different colostrum quality, 5 of these studies categorized colostrum Ig concentration as high, medium, or low, 6 studies categorized colostrum quality by the number of milkings (e.g., first, second, or third milking) with further analysis of the Ig concentration, 1 compared 5 levels of colostral Ig concentration, 1 mixed different amounts of maternal colostrum with colostrum powder, and 1 compared unfermented and fermented colostrum. Among the 5 studies that evaluated high or medium or low Ig concentration and 6 studies that evaluated the number of milkings, 7 reported the average colostrum IgG or IgG_1_ concentration that were compared which is shown in Fig 5. Thirty-two cohort studies evaluated the associations of colostrum quality with passive immunity transfer in calves. Sixteen of these studies assessed quality by directly measuring colostrum IgG concentration, and 2 measured gamma-globulin concentration or Ig concentration by electrophoresis. Others indirectly measured colostrum IgG concentration including 8 studies used a colostrometer, and 6 used Brix refractometer. One cross-sectional study explored colostrum quality as colostrum Brix %.

#### Timing of first colostrum feeding

**Fig 6:**
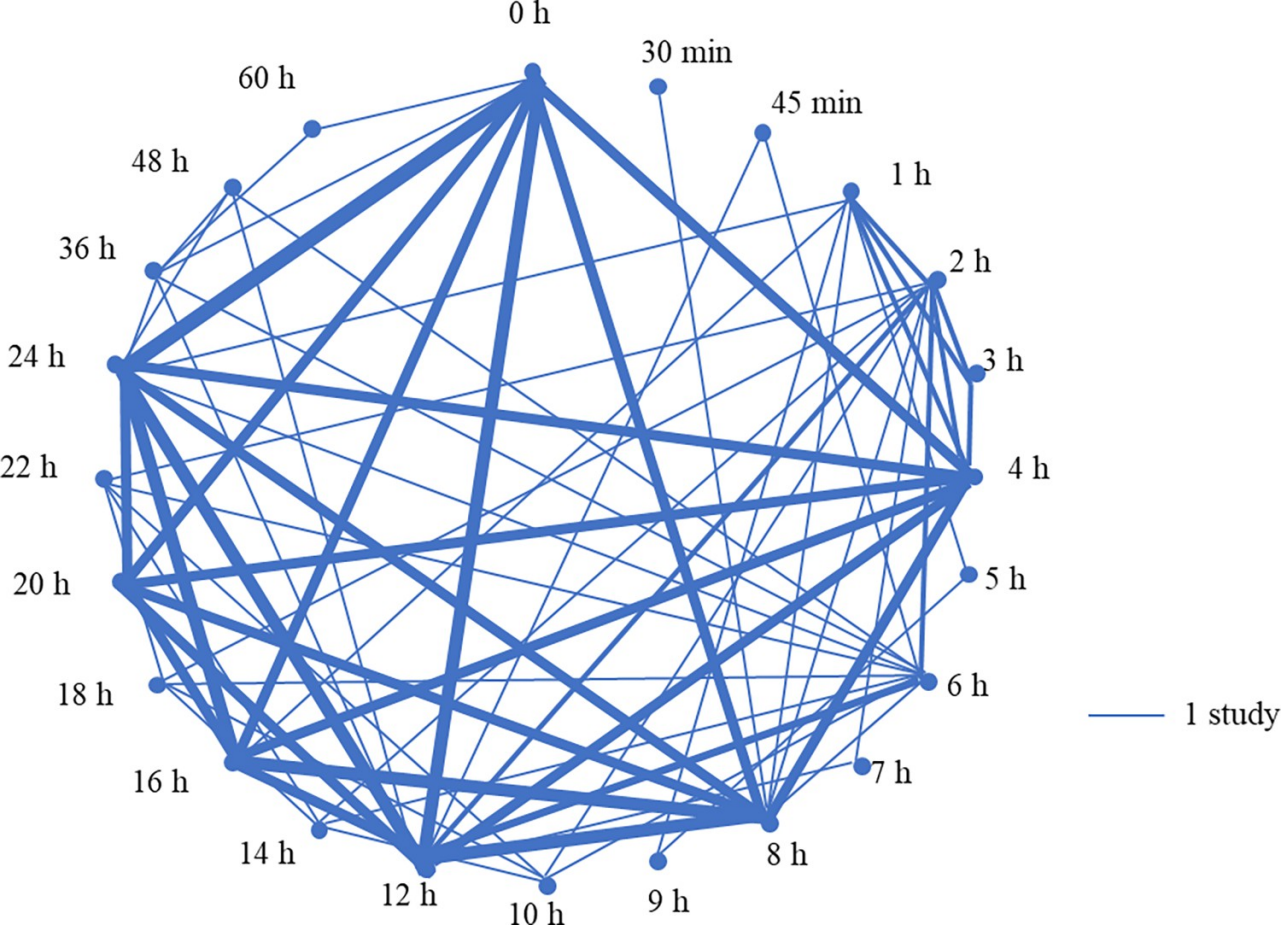
Schematic summary of the times compared in 21 controlled studies on the timing of first feeding of colostrum. Three studies were excluded since the timing of feeding ranged over multiple time periods or had an unclear definition. The thickness of lines represents the number of studies conducted between the combinations of treatment groups.

Twenty-one controlled trials assessed the timing of first colostrum feeding after birth (S5 Table: https://doi.org/10.5683/SP3/NUOT4Phttps://dataverse.scholarsportal.info/privateurl.xhtml?token=3f440ea7-4e5c-43ff-a36a-8b46da6010ca), where 20 studies controlled the timing of feeding to calves and 1 study compared calves kept together or separated from the dam and assessed the relationship between the timing of first feeding with serum IgG concentration. The treatment groups varied largely between studies as shown in Fig 6. Fourteen cohort studies assessed the timing of the first feeding of colostrum. Eight studies investigated the age or timing of first feeding after birth as a continuous variable, 2 classified the interval into 0.5 to 10 h categories, and 1 used both continuous and categorical variables. Three studies did not report how the data were analyzed but had a short explanation on the result of association between timing of feeding and the outcome measured. Two cross-sectional studies assessed the timing of first feeding.

#### Colostrum source

Fifty-eight controlled trials compared different sources of colostrum (S6 Table: https://doi.org/10.5683/SP3/NUOT4P). Fourty of these studies compared feeding maternal colostrum to feeding CR or CS or mixing CR or CS with maternal colostrum, 4 solely compared different CR or CS products, 3 compared colostrum from the calf’s own dam or pooled colostrum, and 1 compared feeding colostrum from its own dam, different dam, or pooled. Two studies compared colostrum from different dam breeds, but the calves were the same breed. Two studies compared colostrum from dams fed different diets and fed to calves born from dams fed the same diet, in addition to the effect of calves born from dams fed different diets. One study compared maternal colostrum to mixing maternal colostrum with milk. One study compared colostrum from heat-stressed or cooled dams fed to calves born from dams in thermoneutral condition, in addition to the effect of calves born from dams heat-stressed or cooled. Other controlled studies explored the use of dried colostrum protein concentrate, lyophilized colostrum protein concentrate, freeze-thawed colostrum, and low-fat fortified colostrum powder. Two cohort studies assessed the source of colostrum including 1 that compared colostrum from dams at different lactations. One cross-sectional study compared colostrum from its own dam, pooled, CR, or a combination.

#### Prepartum diet

Thirty-one controlled trials evaluated the effect of differing pre-parturient diets fed to dams on transfer of passive immunity in their calves (S7 Table: https://doi.org/10.5683/SP3/NUOT4P). Ten studies compared different levels of minerals in the diet, with 3 on selenium, 2 on a mixture of zinc, copper, and manganese, 1 on dietary cation-anion difference (DCAD), 1 on iodine, 1 on copper, 1 on zinc, and 1 on a mixture of iodine, selenium, and cobalt. One study compared different forms of selenium, feeding either selenium yeast or sodium selenite. One study compared the level of DCAD combined with the duration feeding the diet. One study compared the level of DCAD combined with the dietary calcium concentration. Six studies investigated dietary energy including 3 comparing different levels of dietary energy, 2 comparing different amounts of concentrate fed, and 1 comparing corn and wheat grain as sources of starch. Three studies assessed fat sources, where 2 studies compared different levels of dietary fat, and 1 study compared feeding a diet with or without linseed. Three manipulated vitamin levels including 2 studies on nicotinic acid and 1 on a combination of folic acid, biotin, and vitamin B_12_. Three studies investigated the effect of adding dietary microorganisms including 1 on mannan oligosaccharides, 1 on yeast, and 1 on direct-fed microbials. In addition, 2 studies fed different levels of dietary protein, and 1 added algae to the diet.

#### Heat treatment of colostrums

Twenty-five controlled trials investigated the effect of feeding heat-treated colostrum to calves (S8 Table: https://doi.org/10.5683/SP3/NUOT4P). All compared unheated colostrum to a combination of time and temperature of heating, including 6 studies that heated to 60° C for 30 min, 6 heating to 60° C for 60 min, 1 heating to 60° C for 30 min or 60 min, 1 heating to 63° C for 15 sec, 3 heating to 63°C for 30 min, 1 heating to 63° C for 60 min, 1 heating to 76°C for 15 min and 1 heating to 76°C for 15 sec. Two studies compared non-irradiated colostrum to colostrum irradiated either by electron beam or γ-irradiation. One compared pressure-treated colostrum (400 MPa for 15 min) to colostrum pasteurized at 60°C for 60 min, and 1 compared colostrum heated in different batch pasteurizers, both to 60°C for 60 min, with unheated colostrum. One study assessed thawing colostrum with boiling water or a microwave oven. Three cohort studies assessed the use of heated colostrum, with 2 studies comparing pasteurized and unpasteurized colostrum without information on the temperature or the duration, and 1 not describing the method of heat treatment. One cross-sectional study compared pasteurized to unpasteurized colostrum.

#### Thermal stress or calving season

Seven controlled trials assessed the effect of heat or cold stress on passive immunity transfer in calves (S9 Table: https://doi.org/10.5683/SP3/NUOT4P). Three studies evaluated the effect of cooling heat-stressed pregnant dams while 2 compared dams kept in shade to dams kept in shade with fans and sprinklers, and 1 compared dams kept without sprinklers or fans to dams with sprinklers and fans. One study compared the combined effect of heat stress and cooling in both pregnant dams and calves, where dams were provided shade only or shade with fans and soakers and calves were either allocated to an environment with a shade in an open-sided barn with curtains or a cooler environment with shade and fans. Specific to calves, 2 studies evaluated the impact of cold stress on passive immunity transfer, where 1 compared newborn calves wiped dry and transferred to a heated calf room to newborn calves not dried and transferred to unheated room, and 1 compared calves immersed in water at 15°C to 17°C or 35°C to 37°C. One study evaluated the impact of heat stress on passive immunity transfer, where calves kept in shade only, shade with evaporative cooling system, or hutch were compared. Eighteen cohort studies investigated the association between calving season and transfer of passive immunity with a large variety of definitions of seasons. Sixteen compared among 2 or more seasons and 2 used climatological indices including average temperature humidity index (THI). One cross-sectional study compared calving season among winter, spring, and summer.

#### Dam parity

Twenty-two cohort studies assessed dam age or parity. Fourteen studies assessed the number of parities or lactations, 6 compared primiparous and multiparous dams, and 2 reported using dam age or parity but showed no details on how these variables were analyzed. Two cross-sectional studies assessed dam age or parity.

#### Dam breed

Seven cohort studies explored dam breed. Three used Holstein breed, 2 used crossbreeds of Holstein, 1 compared purebred and crossbred Jersey, and 1 included Belgian Blue White and Friesian Black-Pie breeds. One cross-sectional study assessed dam breed, comparing Jersey to Friesians.

#### Dam vaccination

Six controlled trials assessed the effect of vaccinating dams before calving (https://doi.org/10.5683/SP3/NUOT4P). All studies used killed vaccines. Two compared dams unvaccinated or vaccinated with a combination of rotavirus, coronavirus, *E*. *coli*, and *C*. *perfringens*, 2 compared dams vaccinated against *M*. *haemolytica* to unvaccinated dams, 1 compared unvaccinated to vaccinated dams for clostridial diseases, and 1 compared dams vaccinated in early or late gestation in combination with booster shots for foot and mouth disease.

#### Housing the calves with dams

Nine controlled studies kept the calves together with the dam to assess the effect of natural suckling compared to hand feeding (S11 Table: https://doi.org/10.5683/SP3/NUOT4P). One kept calves with their dams for 24 h, 1 for 48 h, 2 for 3 d, 2 for 4 d, 1 for 5 d, 1 for 28 d, and 1 for longer than 42 d. Two cohort studies assessed non-separation of calves from the dams with 1 comparing calves separated from their dams to those kept with their dams for 2 d, and 1 investigated the time spent in calving area as a continuous variable and further categorizing it to 0, 0–10, 10–60, and > 60 min. One cross-sectional study investigated the timing of separation of calves from their dam.

#### Method of colostrum feeding

Eleven controlled trials evaluated the method of feeding colostrum (S12 Table: https://doi.org/10.5683/SP3/NUOT4P). Seven studies compared nipple bottle to esophageal tube feeder, 1 compared nipple bottle to bucket feeding, 1 compared nipple bottle, esophageal tube, or natural suckling, 1 compared nipple bottle, esophageal tube, or placing a tube but feeding thereafter by nipple bottle, and 1 compared nipple bottle, esophageal tube, and a combination of both. Six cohort studies assessed the route of feeding colostrum, with 4 including esophageal tube as a method and 2 assessing suckling by leaving the calf with the dam. Three cross-sectional studies evaluated the method of colostrum feeding including nipple bottle, esophageal tube, and others.

#### Colostrum storage

Five controlled trials assessed the storage of colostrum, where 1 compared fresh colostrum to frozen colostrum, 1 compared fresh colostrum with that stored at 4°C, 13°C, or 22°C for 2 d, 1 compared frozen colostrum to colostrum kept at 4°C for no more than 48 h, 1 compared fresh colostrum, frozen colostrum, and cell-free colostrum, and 1 compared frozen to lyophilized colostrum (S13 Table: https://doi.org/10.5683/SP3/NUOT4P). Four cohort studies assessed storage including 1 that compared fresh to frozen or refrigerated colostrum depending on its quality, 1 compared refrigerated and frozen colostrum, 1 explored the use of fresh and frozen colostrum, and 1 investigated the time in the refrigerator from harvest to feeding calves. One cross-sectional study compared different storage as frozen, fresh, or combination of these.

#### Bacterial contamination of colostrums

Four controlled trials evaluated the bacterial load in colostrum, where 1 compared low (fresh frozen colostrum with standard plate count of 3.97 log_10_ cfu/ml) to high bacteria (colostrum kept in 20°C for 24 h with standard plate count of 5.61 log_10_ cfu/ml), 1 compared low (fresh frozen colostrum with standard plate count of 4.59 log_10_ cfu/ml) to high bacteria (colostrum kept in 20°C for 60 h with standard plate count of 8.63 log_10_ cfu/ml), 1 compared low to high bacteria with a cut-off of 100,000 cfu/ml of total bacterial count (TBC), and 1 compared pasteurized to non-pasteurized colostrum and later assessed the relationship with total plate counts and coliform counts with passive immunity in calves (S14 Table: https://doi.org/10.5683/SP3/NUOT4P). Three cohort studies assessed bacterial load in colostrum with 1 investigating total counts of viable bacteria as a continuous variable and 1 evaluating the aerobic bacterial count (cut-off: 100,000 cfu/ml) and coliform count (cut-off: 10,000 cfu/ml) in colostrum on passive immunity. One study explored total plate count and total coliform counts as continuous variables.

#### Extended colostrum feeding

Four controlled trials assessed the effect of extended feeding of colostrum, with 2 studies exploring the use of transition milk (i.e., second to 6^th^ milkings after calving) [1] and 2 studies investigated feeding colostrum for 14 d or longer (S15 Table: https://doi.org/10.5683/SP3/NUOT4P).

#### Dry cow period length

Two controlled trials manipulated dry period lengths, where 1 study compared cows that were dry for 0, 30, or 60 d, and the other study compared a 4 week to 8 week dry period (S16 Table: https://doi.org/10.5683/SP3/NUOT4P). Four cohort studies investigated dry period length including 2 that did not report how the variable was included in the analysis.

#### Calving difficulty

One controlled study assessed hypoxia by comparing calves provided with 10.5% or 21% oxygen for 24 h by face mask (S17 Table: https://doi.org/10.5683/SP3/NUOT4P). Twelve cohort studies assessed the association of calving difficulties with the transfer of passive immunity. Three compared calving with or without assistance, and 2 compared eutocia to dystocia. One study compared assisted to non-assisted calving. Six studies compared among 3 or 4 levels of calving difficulty (e.g., unassisted, easy, hard, or veterinary intervention), including 1 of the studies evaluated respiratory acidosis in calves. Additional 4 cohort studies assessed hypoxia or acidosis in calves, measuring various combinations of blood pH, oxygen, CO_2_, and HCO_3_ concentrations. Two cross-sectional studies assessed calving difficulties including unobserved, unassisted, and multiple levels of assistance.

#### Other intervention or risk factor

No study included the timing of harvesting colostrum after calving to the level of TPI in dairy calves. Three controlled trials included the volume of colostrum produced by the dam as a covariate in their study.

### Outcome definitions

#### Timing of blood sampling

The timing of collection of blood samples from calves was reported as the age of the calf in 79% (n = 203) of the studies and 21% (n = 53) reported the time after colostrum feeding or intervention. Among the studies that sampled calves based on their age, 50% (n = 102) sampled on day 1, 23% (n = 46) sampled on day 2, 14% (n = 28) sampled between 3 and 9 d, 7% (n = 14) between 1 and 7 d, 2% (n = 4) sampled between 1 and 8 d, and the other studies used different age categories but sampled all calves within 8 d of age. Among the studies that sampled calves based on the timing after colostrum consumption or intervention, 87% (n = 46) sampled within 24 h after the treatment, and 13% (n = 7) sampled between 25 h and 48 h after treatment.

#### Methodology to assess TPI

The level of passive immunity in calves were assessed by serum or blood IgG concentration (87%; n = 222), TP concentration (42%; n = 107), GGT concentration (4%; n = 11), and Brix percentage (3%; n = 8). Among the 222 studies which measured blood IgG concentration, 65% (n = 144) used radial immunodiffusion (RID), 18% (n = 41) used enzyme-linked immunosorbent assays (ELISA), 8% (n = 17) did not report the method, 8% (n = 17) used the turbidimetric procedure, and 1% (n = 3) used electrophoresis. Among the 107 studies which measured blood TP concentration, 29% (n = 31) used a refractometer but did not report whether it was digital or optical, 19% (n = 20) used a kit or an analyzer or both, 15% (n = 16) used a digital refractometer, 15% (n = 16) did not report the method, 10% (n = 11) used the biuret method, and 8% (n = 8) used an optical refractometer. Among the 8 studies that measured Brix %, 4 used a digital refractometer, 2 used an optical refractometer, and 2 did not specify.

#### FTPI definition

The proportion of calves with FTPI was measured in 25% (n = 64) of the studies. Most of these studies (63%; n = 40) used the cut-off value of 10 g/L of IgG or Ig, followed by 5 studies using a 5.2 g/dl cut-off for TP, and 3 used TP < 5.5 g/dl. Others used a variety of cut-points of blood IgG, TP concentration, or Brix %.

## Discussion

This scoping review catalogues a wide variety of studies that explored associations between colostrum characteristics and management and the level of TPI. Most studies (76%) were controlled trials, followed by cohort studies (22%). One-third of the controlled trials investigated feeding different sources of colostrum (e.g., CR, CS), followed by pre-parturient diet treatments. A limitation to consider when interpreting the results of this review is that only studies written in English were included in data synthesis and may not represent colostrum practices implemented worldwide. A few studies conducted in non-English speaking countries could have been excluded, since they were written in languages other than English.

Thirty controlled trials investigated the quantity of colostrum at first feeding, with 17 studies specifically on maternal colostrum. Nine of these studies compared different groups of calves fed 2 L or less, and the remaining 8 studies compared calves fed 3.8 L or higher to those fed ≤ 3 L. The latter 8 studies were comparable to each other, including Holstein calves fed colostrum soon after birth and blood IgG concentration measured within 2 d of age in most of the studies. Therefore, further meta-analysis may be performed although the variety of colostrum IgG concentration used among the studies may need to be considered. Few studies provided evidence for the recent guidelines suggesting that calves be fed at least 10% of their body weight at first feeding within 2 h of birth [9], and dairy calves be fed 4 L of good-quality colostrum within 12 h of birth [10]. Observational studies show that colostrum practices on commercial dairy farms may be insufficient, where mean volumes of colostrum fed within 6 h after birth was 3.3 L in Ontario [11], and the mean or median quantity of colostrum at first feeding in a US national study was less than 3 L [12]. A study in New Zealand found nearly half of the dairy farms fed 2 to 2.5 L of colostrum to calves within 24 h, but calves may have already suckled from their dam since it is a common practice to keep calves together with its dam for a longer period [13]. In a survey on seasonal-calving farms in Ireland, 41% of dairy farmers relied on natural suckling and did not know how much colostrum calves consumed at first feeding [14]. More controlled trials should be conducted to provide evidence on the support of current recommendations.

Twenty-four controlled trials investigated the association between colostrum quality and the level of FTPI in calves with a variety of definitions used for “high” and “low” quality of colostrum. High-quality colostrum was defined as IgG concentration ranging from a minimum of 56 to 106 g/L among the studies. Similarly, low-quality colostrum was defined as IgG concentration ranging from a minimum of 18 to 56 g/L, not necessarily defined with the recommended cut-off of IgG < 50 g/L [1, 15]. The wide variety of colostrum IgG concentration used among the studies limits further quantitative analysis. More research may be needed to derive a robust, standard cut-point if one exists or to identify the variables that modify the target under different conditions. Studies show that 21% to 52% of colostrum samples on commercial dairy farms in Canada, Australia, and Ireland had IgG < 50 g/L [16–18]. The proportion of US dairy producers who evaluate colostrum quality increased from 6% (2002) to 53% (2014) [19], with 49% of producers in Ontario [11], and 48 to 71% of producers in different areas of California [20] testing colostrum quality. More producers seem to have become aware of the importance of measuring colostral quality in relation to TPI in calves. However, the timing of collecting colostrum can affect its quality [21], but no study explored the timing of collecting colostrum after calving in relation to the level of TPI highlighting this variable as a knowledge gap.

Twenty-one controlled trials explored the timing of first feeding of colostrum, which varied from feeding immediately after birth to 60 h of age. This wide range does not align with the recommendation to feed colostrum within 2 h of birth [9]. Only 10 studies compared calves fed within 2 h of birth to calves fed between 2 to 6 h of birth, but 5 of these studies were conducted before 1980. Therefore, these studies may not be comparable with each other for a meta-analysis due to the changes that have occurred in management practices through time. More new research should be performed to investigate the effect of prompt first feeding. In Ireland, 84% of producers fed colostrum within 3 h of birth [14], but only 12% of farms in New Zealand fed colostrum within 6 h of birth [13]. The median timing of first feeding was 2 h in a US nation-wide study [12] and 2.5 h in farms in Ontario [11], suggesting that half of calves may be fed in a timely way. As such, for smaller dairy farms where maternity and calf facilities are not staffed 24 h per day, potentially offsetting variables should be investigated with the timing of first feeding (e.g., colostrum volume at first feeding and cumulatively to 12 h of age, and IgG concentration).

Only 4 controlled studies and 3 cohort studies described the association between bacterial load in the colostrum and the level of TPI in calves. A suggested target is TBC < 100,000 cfu/ml [1]. However, only 55% of colostrum samples met the criterion in US dairy farms [22], and only 20% of colostrum samples had both TBC <100,000 cfu/ml and IgG > 50 g/L in Australian dairy farms [17]. Pasteurization is one method to reduce the bacterial load in colostrum, which was investigated in 3 controlled trials included in this review [23–25]. However, other management factors including colostrum storage and cleanliness of feeding equipment could contribute to transfer of bacteria to calves. One study found that 86% of dairy producers took longer than 5 h to store colostrum [14] and another found that more than half of the nipple bottles and esophageal feeders had TBC > 100,000 cfu/ml [26]. Therefore, more research is needed to validate targets for bacterial load in colostrum and to identify potential interacting variables for passive transfer and health outcomes.

Most studies (87%) assessed the level of TPI by measuring calf blood IgG concentration, mainly by RID. Blood TP concentration was measured in 42% of the studies, with a refractometer used in 29% to estimate blood IgG concentration. More than half of the studies (63%) used a cut-off of IgG < 10 g/L to define FTPI. Radial immunodiffusion is recognized as the gold standard for IgG measurement, but studies suggest values measured in RID could be different from those by ELISA [27, 28]. Although a refractometer is practical to quickly estimate the level of TPI on farms, TP may not be the ideal variable to assess TPI in calves fed CR [29]. The difference in testing methods need to be considered, and further synthesis may be required to establish the method with the greatest utility.

## Conclusions

This scoping review identified few studies that supported evidence for the current industrial recommendations on colostrum management practices in dairy calves. The association between colostral quantity at the first feeding and the level of TPI in calves included comparable studies that could be used for quantitative analysis in the future. Colostral quality was not defined uniformly across the studies, which can be difficult to assess in a meta-analysis but may be included as a continuous variable in a meta-regression. Half of the controlled studies on the early timing of feeding colostrum were conducted more than 40 years ago, which could increase the heterogeneity in management practices other than the colostrum. The association between bacterial load in the colostrum and the level of TPI in calves was rarely studied and remains a knowledge gap.

## Supporting information

S1 Checklist Preferred Reporting Items for Systematic reviews and Meta-Analyses extension for Scoping Reviews (PRISMA-ScR) checklist. (DOCX)
